# The role of sialomucin CD164 (MGC-24v or endolyn) in prostate cancer metastasis

**DOI:** 10.1186/1471-2407-6-195

**Published:** 2006-07-21

**Authors:** AM Havens, Y Jung, YX Sun, J Wang, RB Shah, HJ Bühring, KJ Pienta, RS Taichman

**Affiliations:** 1 Department of Periodontics and Oral Medicine, University of Michigan School of Dentistry, 1011 North University Ave., Ann Arbor, Michigan 48109-1078, USA; 2 Department of Pathology, University of Michigan, 1500 E. Medical Center Drive, Ann Arbor, Michigan 48109-0692, USA; 3 Department of Internal Medicine II, Division for Hematology, Immunology, Oncology and Rheumatology, University Hospital, Tübingen, Germany; 4 Department of Urology, University of Michigan, 1500 E. Medical Center Drive, Ann Arbor, Michigan 48109-0692, USA; 5 Department of Ophthalmology, Affiliated hospital of Jiangsu University, Zhenjiang, Jiangsu 212001, China

## Abstract

**Background:**

The chemokine stromal derived factor-1 (SDF-1 or CXCL12) and its receptor CXCR4 have been demonstrated to be crucial for the homing of stem cells and prostate cancers to the marrow. While screening prostate cancers for CXCL12-responsive adhesion molecules, we identified CD164 (MGC-24) as a potential regulator of homing. CD164 is known to function as a receptor that regulates stem cell localization to the bone marrow.

**Results:**

Using prostate cancer cell lines, it was demonstrated that CXCL12 induced both the expression of CD164 mRNA and protein. Functional studies demonstrated that blocking CD164 on prostate cancer cell lines reduced the ability of these cells to adhere to human bone marrow endothelial cells, and invade into extracellular matrices. Human tissue microarrays stained for CD164 demonstrated a positive correlation with prostate-specific antigen levels, while its expression was negatively correlated with the expression of androgen receptor.

**Conclusion:**

Our findings suggest that CD164 may participate in the localization of prostate cancer cells to the marrow and is further evidence that tumor metastasis and hematopoietic stem cell trafficking may involve similar processes.

## Background

Bone metastases are present in approximately 85% of patients who die due to advanced prostate cancer. Tumor cells metastasize to bone via the venous plexus and spread to the axial skeleton, pelvis and spine. Although the pattern of metastasis of some cancers may be explained by anatomy of the efferent venous and lymphatic drainages, anatomy alone does not account for the metastatic pattern of most cancers [1]. Stephen Paget proposed the 'seed and soil' hypothesis in which seeds that represent metastatic cancer cells grow preferentially in the soil of bone matrix as an explanation for the clinical observations that prostate cancers preferentially localize to specific organs [2,3]. These observations suggest that factors in addition to those that arise within the tumor itself contribute to the metastatic fate of cancer cells.

As for metastasis, hematopoietic stem cells also 'home' to the bone marrow. Here the molecular determinants of stem cell homing have recently been determined to be largely dependent on a CXC motif chemokine, stromal-derived factor-1 (SDF-1 or CXCL12), and its receptor, CXCR4. Studies of animals that lack the *CXCL12 *or *CXCR4 *genes (knockouts) are born with marrows devoid of hematopoietic activities, despite the fact that normal fetal liver hematopoiesis occurs [4,5]. Further evidence of the importance of CXCL12 and CXCR4 to stem cell homing includes the observations that progenitor cells and other blood cells move towards gradients of CXCL12, progenitor cell engraftment into nude mice is blocked by anti-CXCR4 antibodies [6] and the migration of hematopoietic progenitor cells from the blood to the marrow is enhanced by the expression of elevated CXCR4 levels [7].

Based upon these observations, we hypothesized that prostate  cancer (CaP) cells may use the CXCL12/CXCR4 axis to home to bone marrow [8-11]. We reported previously that CXCR4 expression is correlated with tumor grade [8], and that CXCL12 signaling through CXCR4 triggers the adhesion of prostate cancer cells to bone marrow endothelial cells [11]. Furthermore, antibodies that recognize CXCR4 block the metastatic spread of the prostate cancer cells to bone in an *in vivo *animal model [9]. Similar findings by others suggest that the CXCL12/CXCR4 axis may play a similar role in the metastasis of other tumors types to the marrow [12,13], including breast cancers [13], melanoma [14], pancreatic, neuroblastoma and renal carcinomas [15,16]. Importantly, our observations in prostate cancer have largely been substantiated by others [17,18].

It is not clear how the CXCL12/CXCR4 axis promotes metastasis. One possibility is that the binding of CXCL12 to CXCR4 activates adhesion molecules that mediate the binding of prostate cancer cells to the bone marrow endothelium. To further elucidate the parallels between metastasis and hematopoietic stem cell homing, secondary data analysis of a large set of DNA microarray data derived from human primary and metastatic human prostate cancer cells published by Dhanasekaran *et al *[19] was performed to identify known and unknown receptors that might regulate stem cell homing. We found that the level of expression of CD164/endolyn/MGC-24v mRNA was greater in bone metastases than in primary tumors.

CD164 was first identified as MGC-24 and cloned as a carrier of a peanut agglutinin-binding site, a tumor-associated carbohydrate marker expressed in human gastric carcinoma cells [20]. The human *CD164 *gene is comprised of six exons (E1-6) that through alternative splicing are expressed as three distinct isoforms; a full-length isoform or CD164(E1-6), the originally characterized form lacking exon 5 (CD164(EΔ5)) and the CD164(EΔ4) variant where exon 4 is deleted [20]. Disulfide-linked 80–85 kDa homodimers are observed, which extracellularly are comprised of mucin domains linked by cysteine-rich motifs [21]. The remainder of the protein is comprised of a transmembrane domain and a 13-amino-acid intracellular region [22]. Like other sialomucins, CD164 is highly glycosylated by both O- and N-linked glycans [23]. Transmembrane forms of CD164/MGC-24 are believed to mediate the adhesive functions [21,24], and are thought to regulate hematopoiesis by facilitating the adhesion of human CD34+ cells to bone marrow stroma [25].

In this report, we demonstrate that the full-length CD164 is expressed by human prostate cancer cell lines under basal conditions. CXCL12 enhances the expression of CD164 mRNA along with alterations in the expression of CD164 protein. Functionally, CXCL12 induced binding to bone marrow endothelial cells or invasion into extracellular matricies can be blocked with monoclonal antibodies that target CD164. Using prostate cancer tissue microarrays, it was noted that the staining intensity for CD164 correlated with increased prostate-specific antigen (PSA) expression. Importantly, CD164 expression in osseous metastasis was greater than that in soft tissue metastasis, most notably in comparison to those found in the liver and lymph nodes. These data suggest that CD164 may play an important role in localizing tumors not only to sites where there are high levels of CXCL12 expression, but also to specific tissue locales. The results of this study suggest that CD164 may play a central role in prostate cancer metastasis.

## Methods

### Primary and secondary antibodies

Antibodies targeting CD164 were generated in the laboratory of Dr. H J Bühring (Tübingen, Germany) and were described in detail previously [23]. These include antibodies targeting the class I CD164 epitope (clone 105A5, mIgM), the class II epitope of CD164 (clone 103B2, mIgG3) and the CD164 class III epitopes (Clones 67D2, mIgG1 and N6B6, IgG2a). In some experiments antibody from the N6B6 clone was purchased from BD PharMingen (San Jose, CA, USA) as well as murine isotype matched monoclonal controls. The control antibodies for these investigations included IgM clone A57H (Dako, Denmark) IgG3 clone 133316 (BD Pharmigen), IgG1 clone 11711, IgG2a clone 20102 (R&D Systems).

### Cell lines

Prostate cancer cell lines (PC3, LNCaP C4-2B, and LNCaP), and bone marrow endothelial cells (HBME) were cultured in RPMI medium 1640, supplemented with 10% FBS, 1% penicillin-streptomycin, and 1% l-glutamine (Invitrogen, Carlsbad CA). PC3 cells were originally isolated from a vertebral metastasis and were obtained from American Type Culture Collection (Rockville, MD). LNCaP cells were originally isolated from a lymph node of a patient with disseminated bony and lymph node involvement. The LNCaP C4-2B cells were derived from the parental LNCaP cell line by serial passage in mice to obtain a more metastatic cell line [30]. The HBME cells were isolated from a Caucasian male and immortalized with SV40 large T-antigen [23]. All cultures were maintained at 37°C, 5% C0_2_, and 100% humidity.

### Secondary data analysis and DNA micro arrays

Secondary data analysis was performed on data published by Dhanasekaran *et al*. [19] as previously reported [8]. Using a 10 K human cDNA micoarray which included ~5,520 known, named genes and 4,464 ESTs, the authors determined the gene-expression profiles of RNA obtained from more than 50 normal and neoplastic prostate specimens [19]. Supplementary information from Dhanasekaran *et. al*. were imported into a Microsoft Excel database and data clustered into normal associated tissues (NAP), benign hyperplasia (BPH), localized cancer (PCa) and metastasis (Met) [19]. The normalized mean and standard deviation values for CD164 was evaluated for significance with Instat 4.0 (GraphPAD software) using one-way analysis of variance (ANOVA), with the level of significance set at p < 0.05.

To determine which of these genes are responsive to CXCL12 and represent known adhesion molecules, RNA from three independent studies, carried out in triplicate and pooled, was analyzed from CXCL12 treated LNCaP and LNCaP C4-2B cells (200 ng/ml, 2 hours,). The RNA was compared in triplicate against non-treated cells by DNA micro array using the Human genome U133 gene chips (Affymetrix Corp. Santa Clara, CA). Protocols and instrumentation setups, including total RNA samples, hybridization to the human micro arrays, washing, staining, and scanning were performed as recommended (Gene Chip Micro arrays; Affymetrix, Santa Clara, CA) by the University of Michigan Dental School Microarray Facility. The resulting arrays were scanned (HP Gene Array Scanner; Hewlett Packard, Palo Alto, CA), and data analysis performed first with the accompanying software to obtain average difference intensities (Gene Chip Expression Analysis Software, ver. 3.3; Affymetrix). Later, average gene intensity values were determined using a software package (DNA-Chip (dChip version 1.1)) [26] in consultation with the University of UMCCC Microarray Core Facility.

### RT- and *Q*RT-PCR

The prostate cancer cell lines at confluence were transferred to serum free media, and stimulated with CXCL12 at 0 or 200 ng/ml for 0.5–8 h. RNA was recovered using Trizol^R ^according to the directions of the manufacturer (Invitrogen, Carlsbad, CA). RNA integrity and purity was evaluated by electrophoresis with ethidium bromide and absorbance at A_260_/A_280_. 1.0 μg RNA, 10X RT buffer (1X RT buffer: 50 mM Tris, pH 8.3, 50 mM KCl, 8.0 mM MgCl_2_, and 10 mM dithiothreitol), 25 mM dXTP mix (25 mM of each dXTP (ACGT)), 3.0 μg oligo d(T), and 2.5 U Reverse Transcriptase (M-MLV Reverse Transcriptase, Invitrogen, Carlsbad, CA) were incubated at 38°C for one hour. Amplification primers were designed using PrimerExpress™ software (Applied Biosystems, Foster City, CA) to cross intron/exon boundaries. Cross-reactivity was determined using BLAST [27], those with the lowest potential were synthesized. One-fifth of the double stranded product was then mixed with 10X Taq/RT buffer (1X Taq/RT buffer: of 10 mM Tris, pH 8.3, 50 mM KCl, 1.5 mM MgCl_2_, 0.01% gelatin, and 2.0 mM dithiothreitol), 1 mM dXTP mix, 500 ng of each sense and antisense oligonucleotide, and 2.5 U Taq polymerase (AmpliTaq DNA Polymerase; Perkin Elmer Cetus, Norwalk, CT). Five pairs of forward and reverse primers were designed to identify the alternatively spliced transcripts of the *CD164 *gene and are presented in Table 1[20]. The samples underwent thermal cycling at 94°C for 1 min, 60°C for 1 min, and 72°C for 2 minutes for 35 cycles, followed by a 10-min extension at 72°C (Perkin Elmer Cetus DNA thermal cycler). The PCR product of PC3, LNCaP C4-2B, and LNCaP cell lines were analyzed on 12% polyacrylamide gels and ethidium bromide stained.

Real time-PCR was also performed using random hexamers and 15.0 μl of SYBR^® ^Green PCR Master for quantitative, two step real-time RT-PCR (Applied Biosystems, Foster City, CA) with 100 nM of the E1 and E2 primers (Table 2) and 2 μl of the RT product in a total volume of 30 μl. The 2^nd ^step PCR reaction (95°C for 30 seconds, 60°C) was run for 40 cycles after an initial single cycle of 95°C for 15 minutes to activate the Taq polymerase. The PCR product was detected as an increase in fluorescence using an ABI PRISM 7700 instrument (Applied Biosystems, Foster City, CA). The mRNA levels were expressed as relative copies (% control) normalized against 18S mRNA (Ambion QuantumRNA™ 18S Universal Primers) and a standard curve constructed from serial dilutions of a purified CD164 cDNA fragment cloned by classical PCR.

### Tissue microarray and immunostaining

High-density tissue micro arrays were constructed from clinical samples obtained from a cohort of over 600 patients, who underwent radical retro pubic prostatectomy at the University of Michigan as a primary therapy (i.e., no preceding hormonal or radiation therapy) for prostate cancer and from materials obtained from the University of Michigan Rapid Autopsy Program. The arrays were provided from the University of Michigan Comprehensive Cancer Center Tissue Core of the Prostate Specialized Program of Research Excellence program as detail previously [28]. Tumors were graded using the Gleason grading system and examined to identify areas of benign prostate, prostate cancer and bone metastasis. The formalin-fixed, paraffin-embedded tissues were deparaffinized and placed in a pressure cooker containing 0.01 M buffered sodium citrate solution (pH 6.0), boiled and chilled to room temp for antigen retrieval. The slides were incubated overnight at room temperature with anti-human CD164 antibody (BD Biosciences, San Jose, CA) diluted 1:100 (10 mg/ml), IgG2a (Clone 20102, R&D Systems, Minneapolis, MN). For PSA levels, antigen retrieval was performed in citrate buffer at pH 6.0. For AR staining, antigen retrieval was performed with EDTA at pH 8.0 in a pressure cooker. Polyclonal antibodies to PSA (1:2000 dilution, Dako Cytomation, Carpenteria, CA) and a monoclonal AR-clone AR 441 (1:50 dilution, NeoMarkers, Fremont, CA) were used for staining as previously reported [29]. A streptavidin/biotin detection method with 3,3'-Diaminobenzidine Tetrahydrochloride (DAB) was employed for signal detection and Harris hematoxylin was used as a counter-stain. Digital images were then acquired with the BLISS Imaging System (Bacus Laboratory, Lombard, IL). Immunostaining intensity was scored by a genito-urinary pathologist: as absent (1), weak (2), moderate (3) or strong (4). Scoring was performed in a blinded fashion using the Profile web based telepathology system without knowledge of overall the tumor grade, tumor size or clinical outcome [30,31].

### Cell-cell adhesion assays

Prostate cancer cell lines were labeled with Vybrant CFDA SE Cell Tracer Kit, (Molecular Probes, Inc., Eugene, Oregon) for 30 min in RPMI according to the recommendations of the manufacturer and washed, and rested for 30 min. CXCL12 pretreatment was performed by incubating the CaP cells with PBS or 200 ng/ml CXCL12 (or 200 ng/ml CXCL12 that had been boiled for 15 min as a negative control), for 30 min at 37°C. Anti-CD164 mAb (N6B6) or an IgG2a isotype matched control mAb (R&D Systems) were added at 0 and 50 μg/ml to block adhesion. The prostate cancer cells (1 × 10^5^) were then directly deposited onto confluent HBME monolayers and cell-to-cell adhesion was preformed for 30 min at 37°C. Adherence was quantified in a 96-well fluorescent plate reader (IDEXX Research Products, Westbrook, ME), and compared to input fluorescent levels. Data are presented as percent change +/- standard deviation compared to non-treated controls.

### Invasion of CaP cells

Cell invasion into a reconstituted extracellular matrices costing of Matrigel™ overlaid on 8 μM pore sized in polyethylene terephthalate membranes was performed in dual chambered invasion plates (BD Biosciences, San Jose, CA). Test cells were placed in the upper chamber (1 × 10^5^cells/well) in serum free RPMI containing 0.1% BSA, and PBS or 200 ng/ml CXCL12 was added to the top or bottom chambers. Spontaneous invasion was compared to invasion supported by a CXCL12 gradient [32]. Anti-CD164 mAb (N6B6) (BD PharMingen, San Jose, CA, USA) or an IgG (R&D Systems. Minneapolis, MN, USA) control was added at 50 μg/ml to both chambers to block invasion. At the termination of the assay (24 h), the chambers were removed and 40 μl of 3-(4,5-dimethylthiazol-2-yl)-2,5-diphenyltetrazolium bromide (MTT; 5 mg/ml, Sigma, St. Louis, MS) was added to the top well and 80 μl of MTT added to the bottom wells, and incubated for a further 4 h at 37°C. After completely removing residual medium or cells from the top chamber, the purple residues attached to the bottom of the invasion chambers and those residues in the invasion matrix and adherent to the bottom of the upper chamber were released with 1 ml isopropanol (Sigma). The invasion chambers were rocked for 30 min at a medium speed, and 100 μl from each well read on a multi-well scanning spectrophotometer (Molecular Devices Corp. Sunnyvale, CA.) at OD_450_.

### Immunohistochemistry

PC3 and LNCaP C4-2B cells were cultured in Lab-Tek II 4-chamber slides (Nalge Nunc International, Naperville, IL, USA) at 5 × 10^4 ^cells/chamber. After 24 h the cells were incubated with PBS or CXCL12 (200 ng/ml) for 2 h, then rinsed three times with ice cold-PBS, fixed in 4% paraformaldehyde for 25 min at room temperature, washed and endogenous peroxidase activity quenched with 75 mM NH4Cl and 20 mM Glycine in PBS at room temperature for 10 minutes. Thereafter, the cells were rinsed with PBS three times. Primary antibody incubations at a 1:50 dilution in PBS using the N6B6 clone or an IgG2a matched isotype control (clone 20102, R&D Systems) for 1 h at room temperature. Antibody detection was performed using an HRP-AEC staining kit using anti-mouse biotinylated antibodies (R&D Systems), and counter stained with hematoxylin (Sigma).

### Statistical analysis

Statistical differences between the means for the different groups were evaluated with Instat 4.0 (GraphPad Software, Inc. San Diego, CA) using one-way analysis of variance (ANOVA), with the level of significance at P < 0.05. All *in vitro *experiments were repeated two to three times with triplicate samples.

## Results

To identify those molecules that are responsible for the CXCL12-induced changes in adhesion and invasion, we examined the patterns of prostate cancer gene expression in human tissues. A secondary data analysis was performed on data published by Dhanasekaran *et al*. [19], as reported previously focusing on known adhesion molecules [8]. For our investigations, a three-step query was used to select genes that were expressed differentially between the benign hyperplasic and normal associated tissues (BHP/NAP) vs. prostate cancer tissue, BHP/NAP vs. metastatic tissue, and prostate cancer tissue vs. metastatic tissue. Discrimination among the selected genes [33] initially produced ~200 candidates that exhibited a significant (at least 3-fold) change in expression in all three queries (Table 3). To determine which of these adhesion genes were responsive to CXCL12, RNA from LNCaP and LNCaP C4-2B cells treated with CXCL12 (200 ng/ml for 2 h) were compared to RNA from untreated cells using a microarray analysis. Adhesion molecules that exhibited a significant change in mRNA expression in response to CXCL12 treatment included the genes that encode CD164 and α_v _integrin (Table 3). Since the expression of CD164 and the α_v _integrin were both elevated in human CaP disease, and increased in response to CXCL12 stimulation, they were chosen for further investigation. The role of CD164 is described herein, where as the regulation of the α_v _integrin in association with the β_3 _integrin in prostate cancers is described elsewhere (Sun *et al*., submitted).

**Figure 2:**
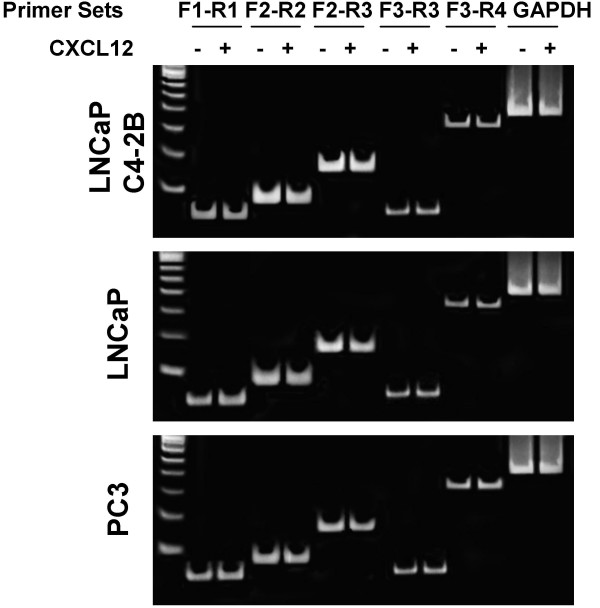
**CaP cells express full length CD164 mRNA**. RT-PCR detection of CD164 expression by LNCaP C4-2B, LNCaP (L) and PC3 (P) cells under basal (-) and CXCL12 (+) stimulated conditions (CXCL12 200 ng/ml at 2 h). Primers F1-R1 were used to identify the CD164 coding exons 1-2, F2-R2 identify exons 2-3 and F3-R3 identify exons 2-5. After RT-PCR, the amplicons were identified using polyacrylamide gels. A molecular weight ladder is shown to the left of each figure that corresponds to differences in 100 base pairs. The data demonstrate that full length CD164 mRNA is express by CaP cells.

Several alternatively spliced variants of CD164 are known to be derived from six transcribed CD164 exons. To determine which of the CD164 transcripts are made by CaP cells, and to verify the microarray data, RNA was collected from PC3, LNCaP, and LNCaP C4-2B cells that were cultured in the presence or absence of CXCL12. For these investigations, all 6 exons of the full-length transcript were examined using reverse transcription-polymerase chain reaction (RT-PCR). Sequencing of the PCR-amplified products verified that the amplicons were derived from full-length transcripts under basal conditions (Figure 2). All of the prostate cancer cells expressed mRNA coding for the full length CD164, and none of the alternatively spliced forms were observed. Cells stimulated with CXCL12 also expressed mRNA for CD164, but once again, none of the alternatively spliced transcripts were observed (Figure 2).

**Figure 3:**
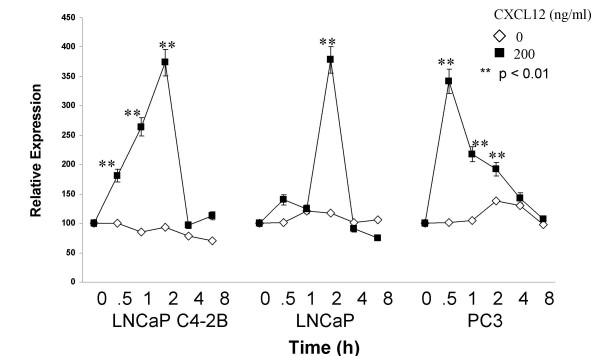
**Time course of CD164 mRNA expression by CaPs**. *Q*RT-PCR was used to detect changes in CD164 expression by CaP cells following CXCL12 stimulation over time for LNCaP, LNCaP C4-2B, and PC3 cells. The data show that LNCaP and LNCaP C4-2B respond to high concentrations of CXCL12 by increasing CD164 mRNA expression. PC3 cells increase CD164 expression to moderate concentrations of CXCL12 (20 ng/ml), but at higher CXCL12 doses (200 ng/ml) down regulate expression.

**Figure 4:**
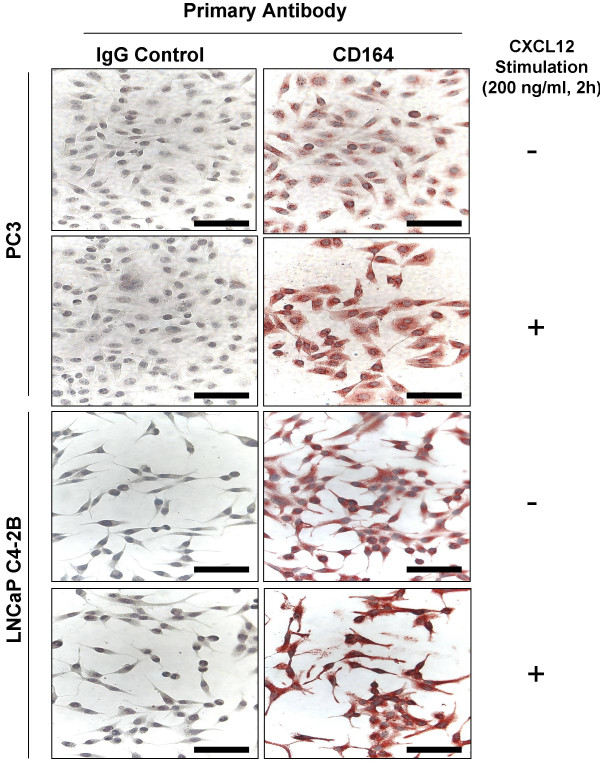
**Expression of CD164 in prostate cancer cell lines is responsive to CXCL12**. To determine if the prostate cancer cells express CD164 protein, and whether its expression was altered by CXCL12, PC3 and LNCaP C4-2B cells were treated with PBS or 200 ng/ml CXCL12 for 2 h, fixed and stained with anti-CD164 mAb or an IgG isotype matched control, and photographed at 40X. Scale bars = 100 μm. The cancer cells demonstatrate perinuclear and cytoplasmic expression of CD164 in nearly all of the cells under basal conditions, and abundantly express CD164 following CXCL12 stimulation.

To evaluate the effect of CXCL12 on CD164, prostate cancer cell lines were treated with CXCL12 and mRNA expression was evaluated using real-time RT-PCR. As before, CD164 mRNA was readily detected under basal conditions in each of the cell lines examined (Figure 3). Under basal conditions the LNCaP C4-2B cells expressed nearly 2 fold more CD164 than PC3 and LNCaP cell lines (not shown). As early as 30 min after exposure to CXCL12, the expression of CD164 mRNA increased in each of the cell lines (Figure 3). In PC3 cells, the enhanced expression of CD164 mRNA at 30 min represented the peak of the response to CXCL12, and the level of CD164 mRNA expression declined thereafter. By contrast, in the LNCaP and LNCaP C4-2B cells, the expression of CD164 mRNA peaked 2 h after exposure to CXCL12 (Figure 3). To determine if the prostate cancer cells express CD164 protein, and whether its expression was altered by CXCL12, PC3 and LNCaP C4-2B cells were treated with PBS or 200 ng/ml of CXCL12 for 2 h. Thereafter, the cells were examined by immunohistochemistry. The number of cancer cells exhibiting perinuclear and cytoplasmic expression of CD164 ranged from 90–100% in PC3 and LNCaP C4-2B cells (Figure 4). Abundant CD164 expression was detected in the cytoplasm and on the cell surface of the PC3 and LNCaP C4-2B cells following CXCL12 stimulation (Figure 4).

**Figure 5:**
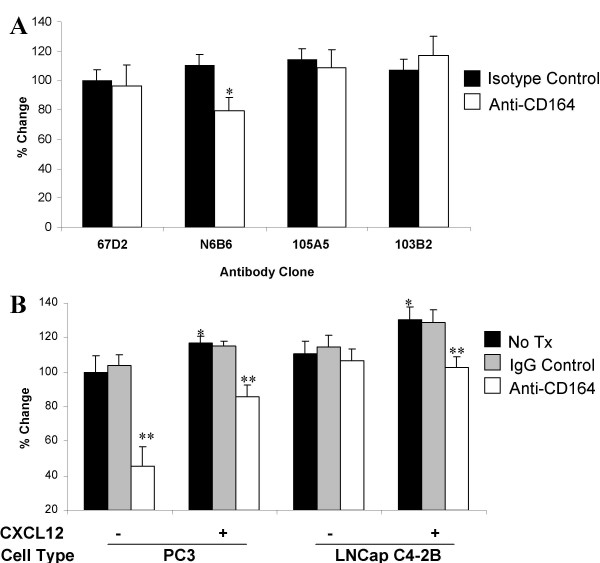
**CD164 regulates CaP cell adhesion to HBME**. PC3 and LNCaP C4-2B cells were labeled with Vybrant CFDA SE Cell Tracer Kit, (Molecular Probes, Inc., Eugene, Oregon) for 30 min and directly deposited on to human bone marrow derived endothelial (HBME) monolayers. CXCL12 pretreatment was performed by incubating the CaP cells with 200 ng/ml CXCL12 for 2 hours. In (**A**) 50 μg/mL anti-CD164 mAb antibodies (or Ig controls) were added to each chamber. Cell-to-cell adhesion for PC3 cells was preformed for 30 min at 37°C and quantified using a 96-well fluorescent plate reader (IDEXX Research Products, Westbrook, ME). Data are presented as percent change +/- standard deviation from non-treated controls for n = 4. In (**B**) the CD164 antibody (N6B6) was used in an adhesion assay with PC3 and LNCaP C4-2B cells treated with either PBS or CXCL12 (200 ng/ml) to block adhesion. * Indicates significant difference from vehicle treated cells (no CXCL12), and ** indicates significant difference from Ig treated controls at p < 0.05. The data demonstrates that CD164 blockade regulates PC3 and LNCaP C4-2B binding to HBME.

We demonstrated previously that activation of CXCR4 by CXCL12 enhanced the adhesion of prostate cancer cells to bone marrow endothelial cells. To determine whether CD164 is involved in CXCL12-induced adhesion, PC3 cells were treated with CXCL12. Binding assays were performed either in the presence or absence of a variety of neutralizing antibodies that recognize various epitopes of CD164 (Figure 5A shown in Figure 5B). Compared to non-stimulated cells, CXCL12 significantly enhanced the adhesion of PC3 cells to marrow endothelium by 13 ± 2.4 % and LNCaP C4-2B by 15 ± 1.1 %, while the adhesion of CXCL12 stimulated cells was inhibited by the class III epitope monoclonal antibody (clone N6B6) (Figure 5A). Similar investigations using the N6B6 antibody on CXCL12 stimulated, or non-stimulated CaPs (PC3 and LNCaP C4-2B cells), demonstrated that neutralization of CD164 decreased the binding of prostate cancer cell lines to human bone marrow endothelium; further suggesting that CD164 plays a role in prostate cancer localization to the marrow.

**Figure 6:**
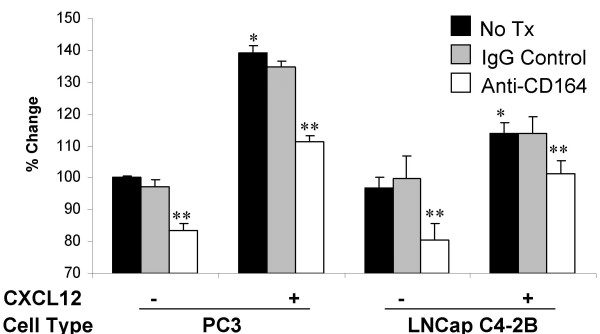
**CD164 regulates CaP cell invasion**. Cell invasion into a reconstituted extracellular matrix was performed using two chambered invasion plates (BD Biosciences, San Jose, CA). PC3 and LNCaP C4-2B cells were placed in the upper chamber (1 × 10^5 ^cells/well) in serum free medium. Spontaneous invasion was compared to invasion supported by a CXCL12 200 ng/ml gradient [32]. Where indicated, IgG control or mouse anti-human CD164 monoclonal antibody (50 μg/ml, Clone N6B6, BD PharMingen) was added at the start of the investigation to each chamber. At the termination of the assay (24 h), the invasion was quantified by MTT. * Indicates significant difference from vehicle treated cells (no CXCL12), and ** indicates significant difference from IgG treated controls at p < 0.05. The data demonstrate that anti-CD164 inhibits invasion of CaP cells

For metastatic prostate cancer cells to establish metastases, they must migrate through the subendothelial matrix after attaching to the endothelium. Migration depends on the availability of chemotactic factors that direct cell movement. To mimic this phenomenon *in vitro*, we explored the effect of CD164 on the ability of cancer cells to invade reconstituted extracellular matrix in response to CXCL12. Migrating cells were introduced into the upper well of an invasion chamber within which a gradient of CXCL12 (0 or 200 ng/ml) was established by placing the chemokine in the lower portion of the chamber. The role that CD164 plays in invasion was probed using a neutralizing anti-CD164 antibody (clone N6B6). As shown in Figure 6, CXCL12 enhanced the invasion of PC3 cells from 21,260 ± 140 to 37,545 ± 459, and LNCaP C4-2B cells from 19,970 ± 639 to 27014 ± 601 cells. In both cases, ant-CD164 antibody blocked invasion of the cells under both basal conditions and in the presence of CXCL12.

**Figure 7:**
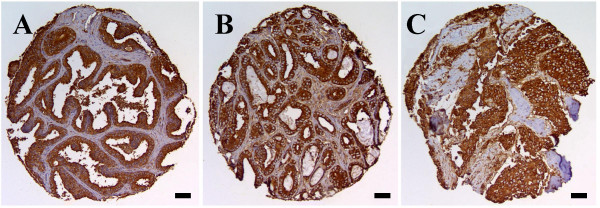
**Human prostate tissue samples stained with CD164**. High-density tissue micro arrays of human CaP tumors were immunostained for CD164, AR and PSA as described in the methods. Immunostaining intensity was scored by a genito-urinary pathologist: as absent (1), weak (2), moderate (3) or strong (4). Scoring was performed blindly using a telepathology system without knowledge of overall the tumor grade, tumor size or clinical outcome [30]. Representative staining of CD164 staining for (**A**) benign prostate hyperplasia (**B**) localized prostate cancer (**C**) metastatic prostate cancer to bone marrow. Correlation of CD164 staining with prostate specific antigen (PSA) staining intensity (**D**), androgen receptor (**E**) and tissue type (**F**). The data demonstrate that CD164 staining intensity increases with increasing tumor grade.

To determine at which stage CD164 is expressed during the progression of prostate cancer, we used an immunohistochemical analysis of clinical specimens of prostate cancer tumors. Staining of microarrays with CD164 monoclonal antibodies revealed that CD164 protein expression was localized to the cytoplasm and that the level of expression ranged from moderate to high in clinically benign and localized prostate cancer tumors (Figure 7A,7B). The level of CD164 expression in malignant epithelia was slightly higher than that in surrounding benign epithelia, while expression in normal epithelium was predominately weak in the cytoplasm and strong within the nucleus. High-grade (Gleason score = 4 + 4 = 8) and low-grade prostate cancer tumors (not shown) exhibited a high level of cytoplasmic expression. Similar staining was observed in bone metastases (Figure 7C). As shown in Figure 7D and 7E, the expression of CD164 was correlated with the expression of prostate specific antigen (PSA) expression but was related inversely to the level of androgen receptor expression (AR). Figure 7F presents data that illustrate the correlation of the intensity of CD164 immunoreactivity and tumor location. Sites that exhibited a high propensity for tumor metastasis and high levels of CXCL12 [9] exhibited intense CD164 immunoreactivity.

## Discussion

Previously, we determined that the CXCL12/CXCR4 chemokine axis is activated in prostate cancers that metastasize to bone [8,11]. To identify which molecules mediate CXCL12-stimulated changes in adhesion and invasion of bone by metastatic cells, we first examined patterns of gene expression in human tissues and cell lines that were stimulated with CXCL12. Our work focused on two genes that play an important role in stem cell localization and the physiology of bone, namely integrin alpha V (CD51) and CD164. Together with CD61, CD51 forms the heterodimer arginine-glycine-aspartic (RGD) motif-dependent vitronectin receptor α_v_β_3_, which is the focus of a parallel report (Sun *et al*., in press); in the present report, we focus on CD164. We present data that demonstrate that CD164 mRNA and protein is expressed by prostate cancer cell lines which are responsive to CXCL12 stimulation. Functionally, antibody blocking of CD164 prevents the binding and migration of prostate cancer cells to bone marrow endothelium and invasion via the extracellular matrix. Importantly, CD164 is expressed in human prostate cancer tissues paralleling the PSA expression, and negatively correlating with AR expression. Notably, CD164 expression in osseous metastasis was greater than that in soft tissue metastasis, in comparison to those found in the liver and lymph nodes – all sites that express high CXCL12 levels[9]. These data suggest that CD164 may play an important role in localizing tumors not only to sites where there are high levels of CXCL12 expression, but also to specific tissue locales.

We found that adhesion and invasion of prostate cancer cells requires the class III epitope, which is recognized by the clone N6B6 antibody. Class I and II epitopes are conformationally independent and are located within the mucin N-terminal domain I [34]. The class I epitope is associated with long-chain sialylated O-linked glycans while the class II epitope is associated with both N- and O-linked glycans. Class III epitopes are conformationally dependent and encompass the cysteine-rich subdomain that links mucin domains I and II [34]. The identity of individual class III epitopes likely depends on the peptide backbone of the protein rather than carbohydrate modifications. Monoclonal antibodies that recognize the carbohydrate-dependent mucin domains of CD164 affect the adhesion and proliferation of hematopoietic precursor cells, yet the CD164 class III epitopes are expressed widely on both hematopoietic and non-hematopoietic cell types [34]. The threeclasses of CD164 epitopes are expressed by a subset of CD34^+ ^hematopoietic progenitor cells and stromal reticular cells from adult bone marrow [35]. In our studies, only monoclonal antibody N6B6 blocked the binding and invasion to bone by prostate cancer cells.

Prostate tumors that develop independence from androgens (hormone-refractory prostate cancer) reflect a transition from a stable to a progressive state of the disease. The development of skeletal metastases may be associated with the progression of prostate cancer towards androgen independence, yet metastasis to bone usually occurs during the late stage of the disease. Recent work revealed that androgen receptor (AR) expression is heterogeneous in cancer patients: individual patients can exhibit both AR-positive and AR-negative tumor populations. Overall, AR expression is down-regulated in hormone-refractory prostate cancer but nearly 41.5% of tumor samples examined expressed some, albeit low (<10%) levels. Previous studies have demonstrated the value of using PSA immunohistochemistry to diagnose metastatic prostate cancer [36,37]. Stein *et al*. [36] demonstrated that most end-stage prostate cancers retain PSA expression. However, AR expression is not correlated with PSA expression, which suggests that PSA expression in late-stage prostate cancers may be driven by mechanisms that are independent of the AR [36]. Our observation that CD164 expression is correlated negatively with AR expression but correlated positively with PSA expression concurs with the aforementioned conclusion and may be useful as a prognostic indicator of androgen-independent tumor growth [38]. Furthermore, the correlation between staining intensity and sites of frequent metastasis and CXCL12 staining is further evidence that CD164 may be involved in tumor localization.

The adhesion of cancer cells to the end-organ vasculature is a crucial step in the metastatic cascade. When combined with organ-specific growth factors, adhesion may have a substantial effect on which site(s) are the target of metastasis [39]. The binding of cancer cells to the microvascular endothelium is believed to require two distinct steps [40]. Initial localization of cancer cells (including prostate cancer cells) to the endothelium appears to be mediated largely by lectins and mucins such as CD164 [41]. Thereafter, activation of integrin receptors and the subsequent development of secure contacts between the cancer cells and endothelium are required. Antibodies that recognize galectin-3 and RGD motif peptides reduce the ability of prostate cancer cells to bind to endothelial tissue [42]. Similar inhibition of binding by antibodies that recognize β1 integrins and CD44 isoforms suggests that integrins participate in the adhesion of cancer cells to endothelial cells [43]. In addition, a mucin-type disaccharide that is expressed on most types of cancer cell (including breast and prostate cancer cells) contributes to adhesion of cancer cells to human bone marrow endothelium [43]. Finally, the expression of hyaluronan, a high-molecular-weight glycosaminoglycan component of extracellular and cell-associated matrices, has been correlated with cancer [44].

As was the case for CD164, we observed that CXCL12 enhanced the transcription and activation of α_v_β_3_. Moreover, the level of α_v_β_3 _expression appeared to be correlated with an increase in tumor grade and malignancy. Unlike CD164, the constitutive expression of this receptor complex represents an inactive state (Sun *et al*., submitted). However, short-term exposure of cancer cells to CXCL12 activates the affinity of prostate cancer cells for α_v_β_3 _ligands in addition to increasing the level of receptor expression. Our experiments revealed that antibodies that recognize CD164 inhibited the binding of prostate cancer cells to human bone marrow endothelium and reduced invasion of the extracellular matrix by prostate cancer cells, irrespective of whether the cancer cells were stimulated by exposure to CXCL12. The abovementioned findings are consistent with the view that metastatic cascades involve several crucial steps, including multiple adhesive events such as initial adhesion and localization of cancer cells to vascular sites and subsequent stabilization between cancer cells and endothelial cells in the face of shear stress. The findings presented in this report suggest that CD164 participates in the initial 'locking' of prostate cancer cells to the endothelium and thereby facilitates the invasion of tissue.

As noted previously, CD164 is both an adhesion receptor on human CD34^+ ^cell subsets in bone marrow and a potent negative regulator of CD34^+ ^hematopoietic progenitor cell proliferation [46]. This is particularly interesting in the light of the results of other studies in our laboratory that revealed that long-term survival of stem cells on osteoblasts *in vitro *(which may be the major source of CXCL12 in marrow) depends on stable adhesion. We demonstrated previously that neutralization of CXCL12 *in vitro *or following intratibial injection *in vivo *inhibited cell growth [45] demonstrated recently that expression of CD164 in myoblast cell lines increased the expression of biochemical markers of differentiation and enhanced the formation of multinucleate myotubes. Similarly, the expression of antisense CD164 or soluble extracellular regions of CD164 inhibited myogenesis. Treatment of cultured C2C12 myoblasts with the enzymes sialidase or O-sialoglycoprotease (both of which destroy functional epitopes on CD164) also inhibited differentiation [45]. These data are consistent with the hypothesis that CD164 may play a rate-limiting role in myogenic differentiation *in vitro *[45]. We noted previously that *in vitro *proliferation and intratibial growth of prostate cancers depends on CXCL12 [8,9]. At present, we are in the process of evaluating whether CD164 plays a similar role in prostate cancer cells. If CD164 does regulate the growth of prostate cancer cells, this would be a particularly attractive therapeutic target for two reasons. First, because CXCL12 up regulates CD164 expression and stimulates prostate cancer cell proliferation and adhesion, therapies designed to disrupt CD164 or the CXCL12/CXCR4 axis could be used to alter tumor progression via several mechanisms. Second, because stem cell homing and niche localization likely depends on multiple and redundant adhesive mechanisms, therapies that target CD164 might have little or no side effects on the host.

In summary, we demonstrated that full-length CD164 is expressed by human prostate cancer cell lines and human prostate cancer tumors. CXCL12 enhanced the binding of prostate cancer cells to bone marrow endothelial cells, and CXCL12 up regulated the expression of CD164 mRNA and protein. Using monoclonal antibodies, we determined that the neutralization of CD164 with antibodies blocked the adhesion of prostate cancer cells to human bone marrow endothelial cells and inhibited subsequent invasion. Collectively, our data suggest that CD164 plays an important role in prostate cancer metastasis and the infiltration of bone marrow by cancer cells.

## Competing interests

The author(s) declare that they have no competing interests.

## Authors' contributions

A.M.H. carried out the molecular and cell culture studies, genetic studies, participated in the sequence alignment and drafted the manuscript. J.Y., designed and performed the adhesion studies, Y-X.S., designed and performed some of the invasion assays, J.W. performed the immunohistochemical studies, R.B.S., directed and evaluated the tissue microarray studies, H.J. B. provided antibodies, K.J.P and R.S.T. participated in the studies design and coordination.

## Pre-publication history

The pre-publication history for this paper can be accessed here:



## Figures and Tables

**Figure 1:**
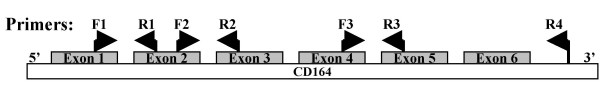
Cartoon of CD164 genomic structure and PCR primer sets.

**Table 1:** CD164 primers and coding regions detected. Five set of oligonucleotide primers were designed to identify differential spliced transcripts derived from of CD164.

**Primers**	**Primer Sequence**
F1	5'-CTCCCGCTGGTCACCACT-3'
F2	5'-GTTAATACTACCTGCTTTTGGATAGAATGT-3'
F3	5'-TTTCCACGGCCACTCCAG-3'
R1	5'-CGCAGCTGTTTCGACCTTC-3'
R2	5'-CCACTTGACAATCACTAACTGTTGAG-3'
R3	5'-GGAGGGCTCAACTGTGGGT-3'
R4	5'-AGTATTTTGGCTTCAGTGAG-3'
GAPDH (F)	5'-GACAACAGCCTCAAGATCATCAGC-3'
GAPDH (R)	5'-AAGTCAGAGGAGACCA-3'

**Table 2:** Coding regions detected in human CD164. Primer sets and regions identified in human CD164.

**Primers Used**	**Regions Detected**	**Size**
F1-R1	Exons 1-2	53 bp
F2-R2	Exons 2-3	82 bp
F2-R3	Exons 2-5	168 bp
F3-R3	Exons 4-5	62 bp
F3-R4	Exon 4-3' untranslated region	327 bp

**Table 3:** Secondary data analysis and DNA micro arrays. Secondary data analysis was performed on data published by Dhanasekaran *et al*. [19] to identify adhesion molecules the expression that are altered during prostate cancer progression. Comparisons were made between normal (norm) tissue/benign prostate hyperplasia (BPH) and cancer (CaP) and metastasis (Met). The normalized mean and standard deviation values were evaluated for significance with Instat 4.0 (GraphPAD software) using one-way analysis of variance (ANOVA) are presented, with the level of significance set at p < 0.05 (*). To determine which of the genes are responsive to CXCL12, RNA was analyzed from CXCL12 treated LNCaP and LNCaP C4-2B cells (200 ng/ml, 2 hours) were compared against non-treated cells by DNA micro array using the Human genome U133 gene chips (Affymetrix Corp. Santa Clara, CA). Later, fold change values were determined using DNA-Chip (dChip version 1.1) [26].

		BPH/Norm Vs CaP		BPH/Norm Vs Met		CaP Vs Met		Fold Change: LNCAP		Fold Change: LNCaP C4-2B	
Gene	Gene Symbol	Mean	S.D.	Mean	S.D.	Mean	S.D.	Mean	S.D.	Mean	S.D.

activated leucocyte cell adhesion molecule	ALCAM	2.34	0.86	1.19	0.76	1.97	0.84	1.04	0.68	1.19	1.47
cadherin 11, type 2, OB-cadherin (osteoblast)	CDH11	2.76	0.20*	1.01	0.27*	2.80	0.27*	-1.09	1.17	-1.38	0.65
cadherin 3, type 1, P-cadherin (placental)	CDH3	3.07	0.27	1.11	0.35	2.87	0.38	-1.19	0.57	-1.06	0.76
cadherin 5, type 2, VE-cadherin (vascular epithelium)	CDH5	1.00	0.37	3.50	1.01	1.00	0.82	-1.03	0.95	-1.12	1.23
CD 3G antigen, gamma polypeptide (TiT3 complex)	CD3G	2.11	0.22	1.12	0.21	1.90	0.27	-1.07	0.68	1.01	0.91
CD 8 antigen, alpha polypeptide (p32)	CD8A	3.24	0.24	1.05	0.24*	3.15	0.25	-1.38	0.60	1.26	0.93
CD 34 antigen	CD34	3.56	0.15	1.20	0.16	2.94	0.16	-1.65	0.85	1.21	0.62
CD 36 antigen (collagen type I/thrombospondin receptor)	CD36	1.96	0.21	1.32	0.33*	1.47	0.37*	1.19	1.07	1.02	0.47
CD 53 antigen	CD53	2.17	0.43*	0.56	0.45	3.84	0.24	-1.05	0.85	-1.02	1.06
CD 68 antigen	CD68	3.31	0.28	1.04	0.25	3.14	0.23	-1.43	0.55	1.02	0.31
CD 79A antigen (immunoglobulin-associated alpha)	CD79A	3.83	0.25	1.27	0.26*	2.98	0.23*	1.19	0.54	-1.25	0.64
CD 151 antigen	CD151	2.10	0.18	0.88	0.16	2.36	0.17*	1.10	0.92	-1.14	0.81
CD 164 antigen, sialomucin	CD164	2.42	0.50	0.68	0.52*	3.58	0.28*	1.32	0.65*	1.95	0.49*
endothelial cell-specific molecule 1	EMAP1	3.01	0.24	1.18	0.30	2.61	0.34*	-3.61	2.14	-2.23	0.36
integrin, alpha 1	ITGA1	1.72	0.60*	0.43	0.70*	4.09	0.34*	-1.05	1.20	-1.20	0.62
integrin, alpha 2 (CD49B, subunit of VLA-2 receptor)	ITGA2	2.06	0.52*	0.53	0.56*	3.93	0.26	1.10	1.84	-1.11	0.97
integrin, beta 3 (platelet glycoprotein IIIa, antigen CD61)	ITGB3	2.46	0.15	1.08	0.15	2.37	0.12	-1.07	1.00	1.06	0.37*
integrin, beta 4	ITGB4	3.22	0.24*	0.62	0.27	4.53	0.21	-1.04	0.73	-1.34	0.59
integrin, beta 5	ITGB5	2.60	0.20	1.18	0.22	2.19	0.19	1.09	1.11	1.01	1.11
integrin, alpha 8	ITGA8	2.09	0.41	0.48	0.32	4.12	0.23	1.14	0.51	1.01	0.87
integrin, alpha V (vitronectin receptor, CD51)	ITGAV	2.10	1.53	0.35	1.40	5.45	0.16*	1.19	0.44*	1.37	0.29
integrin, alpha L (antigen CD11A)	ITGAL	3.48	0.15	0.90	0.16	3.82	0.91	-1.17	0.68	1.24	0.53
protocadherin 1 (cadherin-like 1)	PCDH1	4.36	0.24	0.94	0.28	4.32	0.28	-1.48	0.37	1.17	0.46
platelet/endothelial cell adhesion molecule (CD31 antigen)	PECAM1	3.15	0.25	0.96	0.26	3.26	0.25	1.08	0.83	-1.02	0.67
selectin E (endothelial adhesion molecule 1)	SELE	1.60	1.22	0.33	1.27	4.86	0.30	1.03	0.83	1.10	0.67
syndecan 2	SDC2	2.16	0.34*	0.59	0.37*	3.66	0.17*	-1.12	1.40	1.01	0.76
syndecan 4	SDC4	3.34	0.31*	1.16	0.30	2.95	0.33	1.01	1.21	1.13	0.84
vascular cell adhesion molecule 1	VCAM1	2.34	0.20*	0.91	0.24	2.62	0.23	1.24	1.43	-1.02	0.83
